# The Roles of Monocyte and Monocyte-Derived Macrophages in Common Brain Disorders

**DOI:** 10.1155/2020/9396021

**Published:** 2020-06-04

**Authors:** Mingxia Zhao, Houzhen Tuo, Shuhui Wang, Lin Zhao

**Affiliations:** ^1^Department of Neurology, Beijing Friendship Hospital, Capital Medical University, Beijing, China; ^2^Department of Cardiology, Beijing Anzhen Hospital, Capital Medical University, Beijing, China

## Abstract

The brain is the most important and complex organ in most living creatures which serves as the center of the nervous system. The function of human brain includes controlling of the motion of the body and different organs and maintaining basic homeostasis. The disorders of the brain caused by a variety of reasons often severely impact the patients' normal life or lead to death in extreme cases. Monocyte is an important immune cell which is often recruited to the brain in a number of brain disorders. However, the role of monocytes may not be simply described as beneficial or detrimental. It significantly depends on the disease models and the stages of disease progression. In this review, we summarized the current knowledge about the role of monocytes and monocyte-derived macrophages during several common brain disorders. Major focuses include ischemic stroke, Alzheimer's disease, multiple sclerosis, intracerebral hemorrhage, and insomnia. The recruitment, differentiation, and function of monocyte in these diseases are reviewed.

## 1. Introduction

The human brain is the most sophisticated organ which orchestrates the behaviors of the body and internal organs. Tissue-resident macrophages reside in the brain under normal conditions, including microglia and perivascular macrophages. Under both normal and diseased conditions, the brain will also recruit monocytes to the brain blood vessels and these monocytes further infiltrate into the brain parenchyma. The monocytes, and the macrophages they differentiate into, can help with tissue remodeling and regeneration in diseased conditions [1] or, if uncontrolled, may cause inflammatory damage to the brain tissue and neurons. Monocytes in the host are not homogenous, and the role of the monocytes is complicated and varies significantly in different disease models and with disease progression. Although review articles on the role of monocyte during certain brain disorders exist in literature, the current review provided an overview of multiple brain disorders seeking to find share similarities of monocyte function. A special focus is given on the roles of recruited monocytes and subsequentially differentiated macrophages during major brain disorders. The roles of resident macrophages, especially microglia during brain disorders, are not a major focus in this article.

Monocytes/macrophages are important immune cells with dramatic plasticity which not only plays critical roles during innate immunity but also connects innate immunity and adaptive immunity. In mice, monocytes are classified into two different subsets; one subset demonstrated the following pattern on flow cytometry CX3CR1^low^CCR2^hi^Gr1^+^ and is often regarded as inflammatory or classical monocyte (Ly6C^hi^ population); the other monocyte subset CX3CR1^hi^CCR2^−^Gr1^−^ (Ly6C^low^ population) is normally recruited to noninflamed tissues demonstrating a patrolling behavior and is alternatively named patrolling or nonclassical monocyte [2]. In humans, classical monocytes are defined as CD14^+^CD16^−^ monocytes, while nonclassical monocytes are CD14^low^CD16^+^; in addition, a subset that expresses intermediate levels of CD14 and CD16 also exists in humans. Mice without CCR2 expression have dramatically reduced levels of circulatory classical monocytes but have increased monocyte retention in the bone marrow [3], suggesting that CCR2 signaling is required for classical monocytes to egress from the bone marrow. MCP-3 and MCP-1 are the main ligands for CCR2 which are required for maintaining of normal monocyte number in the circulation. However, CCR2 may not be required for the migration of monocytes to the tissue [4]. The two monocyte subsets also differ in their life span. The life span of nonclassical monocytes is longer than that of classical monocytes in the circulation [5]. The recruit of nonclassical monocyte is reported to be CX3CR1 dependent [6, 7], and this signaling plays an important role during monocyte survival [8]. In a mouse atherosclerosis model, it has been shown that deficiency of CX3CR1 significantly reduces macrophage accumulation and formation of atherosclerotic lesion [9]. The survival of nonclassical monocytes requires a transcription factor nuclear receptor Nuclear Receptor Subfamily 4 Group A Member 1 (Nr4a1) which is also required for the transition of Ly6C^hi^ monocyte to Ly6C^low^ monocyte [10]. After entering tissue, the monocytes could develop into macrophage subsets, among which classically activated macrophage enhances inflammation and promotes pathogen killing while alternatively macrophages inhibit inflammation and promote tissue remodeling.

Having discussed the backgrounds of monocyte, we next reviewed the role of the monocytes during disease onset and progression during common brain disorders. Special attention has been paid to the recruitment, differentiation, and function of different monocyte subsets. It should be noted that a substantial number of literatures exist on the roles of monocyte during brain disorders, and this review does not mean to be all inclusive.

## 2. Ischemic Stroke

Ischemic stroke caused by a focal reduction of cerebral blood flow is one of the most notorious killer in the world, especially in hypertensive individuals [11]. The sudden ischemia in the brain results in loss of cellular integrity and triggered subsequent cell death through either necrosis or apoptosis. The dying cells and debris released trigger a sterile inflammation in the ischemic brain through the release of multiple stimuli. The initial inflammatory responses after stroke are believed to be trigged by pattern recognition receptors (PRRs) recognizing their ligands, including modified or oxidized lipid species, cytoplasmic proteins, DNA, and RNA [12]. These molecules are also called damage-associated molecular patterns, or DAMPs. Atherosclerosis is a major causative factor of ischemic stroke, and monocyte-derived macrophages play a fundamental role during the onset of atherosclerosis [13–15]. The transient or permanent middle cerebral artery occlusion (MCAO) model is currently the most popular rodent model to study ischemic stroke. This model most closely mimics the ischemic stroke in humans [16]. A significant portion of our knowledge about ischemic stroke is obtained through these models.

### 2.1. Monocyte Recruitment

The recruitment of monocyte to the ischemic brain is initiated rapidly after stroke, and monocyte recruitment happens prior to the recruitment of other leukocytes [17]. It is believed the recruitment is mainly triggered by the sensing of DAMPs by resident immune cells like microglial cells, which are activated in a fast fashion [18–20]. In particular, microglia can also sense the release of ATP from damaged cells and migrate to the damaged area; this mechanism has been clearly elucidated [21, 22]. Microglia rapidly release proinflammatory mediators like TNF-*α*, IL-1*β* after ischemic stroke [23, 24], creating an inflammatory environment which facilitated the recruitment of other immune cells including monocytes by activation of the endothelium and release of chemokines.

Studies in stroke models have consistently shown that Ly6C^hi^ monocyte subset is dramatically increased in the brain after stroke [25]. The recruitment of Ly6C^hi^ monocyte to the focal ischemic brain is reported to be CCR2 dependent [26, 27]. Mice deficient in CCR2 displayed reduced Ly6C^hi^ monocyte recruitment to the brain after transient focal cerebral ischemia [26, 27]. Moreover, a selective CCR2 antagonist, INCB3344, prevented the recruitment of Ly6C^hi^ monocyte in a dose-dependent manner [25]. Similarly, mouse deficient in CCR2 ligand MCP-1, which is increased in the brain after stroke, has less phagocytic macrophage accumulation in the infarct area [28–30] suggesting their monocyte origin. Conversely, a study through overexpression of MCP-1 in the brain enhanced with recruitment of inflammatory monocytes [31]. In human patients, the increase of MCP-1 in the cerebrospinal fluid is also noted [12]. Using chimera mice with GFP-labeled bone marrow cells and middle cerebral artery occlusion (MCAO), it was shown that as early as 24 hours post MCAO, bone marrow GFP-positive cells, presumably monocytes, begin to appear in ischemic core and peri-infarct area; these cells later expressed Iba-1 suggesting the transition to microglia [32]. Although it is well established that CCR2-CCL2 axis is essential for the recruitment of monocytes (Ly6C^hi^ subset) to the brain, it is reported that the transmigration of Ly6C^hi^ monocytes into the ischemic brain is a CCR2-independent process in the absence of reperfusion [25]. After recruitment, these Ly6C^hi^ monocytes begin to downregulate the expression of Ly6C while increasing the expression of macrophage marker F4/80 [33]. However, it is interesting that the monocyte-derived macrophages gradually transformed into a status which resembles alternatively activated macrophages by the upregulation of markers like arginase-1 and YM-1 [33].

Comparing studies with the Ly6C^hi^ population, relatively less studies focused on the recruitment of Ly6C^low^ population. Since the development of Ly6C^low^ monocyte requires the transcription factor Nr4a1 [10], mice deficient in this transcription factor have eliminated Ly6C^low^ monocytes during ischemic stroke using a hypoxia-ischemia model [34]. However, the exact signals and adhesion molecules mediating the recruitment of Ly6C^low^ monocyte in the brain during ischemic stroke are still lacking. Recently, using intravital imaging, VCAM and VLA4 signaling has been shown to be involved in the recruitment of Ly6C^low^ monocytes to the brain in an infection model. It remains to be tested whether the same mechanism applies to a stroke model [35]. It should be noted that the Ly6C^hi^ monocyte switches to Ly6C^low^ monocyte after recruitment [36], and this process has also been noted in the mouse MCAO model [37]. Accumulation of Ly6C^low^ monocyte was absent in the brains of CCR2-deficient mice, but not in Nr4A1 chimeric mice; although they lack of Ly6C^low^ monocytes in the circulation, such studies suggest a local transition of Ly6C^hi^ monocyte into Ly6C^low^ subset [37].

### 2.2. Monocyte Functions

The acute onset of stroke leads to an inflammatory environment in the ischemic core, and this sterile inflammation recruits inflammatory cells [19, 33]. The inflammation in the brain after ischemic stroke is a double-edged sword; on the one hand, the inflammation worsens the outcome and is associated with serious outcomes during the early stage; on the other hand, recruited monocytes are required for the initiation of tissue remodeling and recovering at a late stage [33, 38]. Considering the contrasting role of monocyte at different stages, the exact role of monocyte during ischemic stroke may differ.

Although Ly6C^hi^ monocytes are recruited as inflammatory monocytes, however, the majority of literature suggested a protective role of Ly6C^hi^ monocyte in stroke progression models by differentiation into anti-inflammatory macrophages [25, 38]. There are accumulating evidences proving that the Ly6C^hi^ monocytes recruited in different tissues can further differentiate into both M1 and M2 macrophages. Using flow cytometry and immunocytochemistry, it was reported that monocyte infiltration reached maximum at 3 days and gradually demonstrated both proinflammatory and anti-inflammatory phenotype at 7 days but was eventually dominated by anti-inflammatory type. Moreover, blocking CCR2-mediated monocyte recruitment diminished the anti-inflammatory effect and inhibited the long-term behavioral recovery [38]. These results suggest that the Ly6C^hi^ monocyte is crucial for an anti-inflammatory effect at later time points. Deficiency of CCR2 chemokine MCP-1 in mice has no effect on microglia activation seen at a very early stage although with less monocyte recruitment [29]. Moreover, remote postischemic conditioning can benefit stroke recovery by promoting circulating monocytes to proinflammatory subset. Adoptive transfer of CCR2-deficient monocytes failed to provide protection suggesting the protective role of Ly6C^hi^ monocytes [39].

Early recruited peripheral monocytes/macrophages are predominantly Ly6C^hi^ cells that become M1 tissue macrophages in the stroked hemisphere [12]. However, in contrast to this concept, there is a report showing that microglia/macrophages initially displayed M2 phenotype as shown by increased CD206 expression in Iba^+^ cells after ischemic stroke, and these early M2 macrophages are protective of neurons presumably by scavenging the debris from dead cells, gradually converted into M1 phenotype in peri-infarct regions [40]. However, this study did not distinguish the microglia and macrophages.

Little knowledge is in literature when it comes to the role of Ly6C^low^ monocyte subset during ischemic stroke. It is found that with the depletion of Ly6C^low^ monocyte, no much differences were found in terms of total infarct size, cell loss, atrophy, and the number and activation of microglia/macrophages at the lesion site [34]; the authors suggested that Ly6C^low^ monocyte plays redundant roles in the progression and recovery of ischemic stroke. This result is in contrast to the role of Ly6C^low^ monocytes during clearance of vascular amyloid beta (A*β*) in Alzheimer's disease model [41], and the repair of myocardium after ischemic damage [42] and maintaining anti-inflammatory condition in an atherosclerosis model [43].

## 3. Alzheimer's Disease

Alzheimer's disease (AD) is a common neurodegenerative disorder which mainly affects aged people. The disease is also a major cause of dementia [44]. Extracellular A*β* deposition and intraneuronal aggregates of hyperphosphorylated tau (neurofibrillary tangles or NFTs) are the hallmarks of Alzheimer's disease. Patients with AD often have A*β* deposition within the cerebral vasculature. The depiction of A*β* in the brain has been reviewed [45]. Although the exact mechanisms of A*β* during the pathogenesis of AD is still unknown, the detrimental role of A*β* has been well accepted [46]. Transgenic mice overproducing mutant amyloid precursor protein (APP) have been used as a model to mimic human accumulation of A*β* [47]. The mouse models expressing human tau protein have also been used to study the pathogenesis of AD [48]. Using these mouse models, it has been shown that progression of AD often associated with AD-induced inflammation [49, 50] and activation of microglia [51]. In particular, it has been proved that NALP3 inflammasome is a sensor for A*β* which resulted in endocytosis of A*β* and release of proinflammatory cytokines like IL-1*β* [49]. Inflammation during the progression of AD has been extensively studied in recent years; the involvement of multiple cells and molecules was reviewed in [52]. Inflammation causes activation of endothelium and recruits monocytes [45]. These monocytes can develop into macrophages in the AD brain which are critical for restriction of senile plaque formation [53].

### 3.1. Recruitment of Monocyte

Progression of AD causes inflammation and triggers recruitment of leukocytes including monocytes [54]. GFP-expressing bone marrow chimeric mice have been used to study the role of bone marrow cell recruitment to the AD brain [55]. The APP/PS1-dE9 mouse model of AD displayed more GFP-positive cell recruitment comparing to control mice suggesting AD can promote recruitment of bone marrow-derived leukocytes [55]. Using GFP-expressing bone marrow chimeric mice expressing APP, it was shown that the number of GFP-positive cells demonstrating amoeba morphology was significantly increased compared with age-matched control in the brain of aged mice, but not young mice [56]. The expression of monocyte chemokine CCL2 was also shown to be elevated in AD brains [57]. Lack of CCL2 receptor CCR2 leads to lower monocyte accumulation and is associated with higher brain A*β* levels [54]. Another study showed that AD mice deficient in CCR2 accumulate A*β* earlier and die prematurely with accelerated AD-like disease progression [58]. Overexpression of monocyte chemokine CCL2 results in increased microglial accumulation around plaques [59]. The monocytes recruited are likely the precursors for microglial cells as has been showed before [53, 55].

The adhesion molecules required for the monocyte recruitment during AD are being studied; an early study showed that different cellular and substrate adhesive molecules including integrins, as well as their ligands, are present in classical plaques [60]; however, the detailed functions are not very clear at that time. An in vitro study showed that receptor for advanced glycation end products (RAGE) and platelet endothelial cell adhesion molecule 1 (PECAM-1) is involved during the A*β*-induced monocyte recruitment [61]. The transmigration is reported to be protein kinase C dependent [61]. VLA4 have been shown to mediate leukocyte-endothelial interactions in cerebral vessels, blockade of which was shown to improve memory in an AD mouse model [62]; however, this paper did not focus specifically on monocytes.

Not only Ly6C^hi^ monocyte, it has been shown by intravital microscopy that Ly6C^low^ monocytes similarly possess the ability to scavenge A*β* from the A*β*-positive veins [41]. These crawling monocytes can remove the A*β* from the brain and send it back to circulation. Deletion of Ly6C^low^ monocytes caused enhanced deposition of A*β* in the brain [41].

### 3.2. Monocyte Functions during AD

The mechanisms for monocyte to clear A*β* likely involve multiple receptors, including Fc*γ* receptors, scavenger receptor A (SCA), CD36, RAGE, and low-density lipoprotein receptor-related protein (LRP), see review [45]. It should be noted that although monocytes have the ability to clear A*β* from the brain; the ability seems inefficient in monocytes from AD patients [63]. One possible reason is that monocyte characteristics may be altered in patients with AD. It is found that monocyte-derived macrophages from control populations are more capable of phagocytizing A*β* and getting rid of it than monocyte-derived macrophages from AD patients [63]. However, the mechanisms for this change is not very clear. Zaghi et al. have shown that monocytes from AD patients are not only less effective in the phagocytosis of A*β* but also transport A*β* from neurons to blood vessels and release fibrillar A*β* after apoptosis, contributing to cerebral amyloid angiopathy [64]; these studies highlighted the complexed role of monocytes in real AD patients.

### 3.3. Utilizing Monocyte as Therapeutics

Considering the beneficial roles of monocytes, there are studies trying to utilize this cell type for clearance of A*β* in AD patients. A study expressed neprilysin on leukocytes using the 3xTg-AD mouse model of Alzheimer's disease and showed that the expression of this A*β*-lysing enzyme can reduce the soluble brain A*β* peptide levels. Moreover, such treatment did not induce significant accumulation of monocytes in the brain [65]. Monocyte can develop into microglia during AD progression, and it was shown that these microglial cells differentiated from BM-derived progenitor cells are more efficient in phagocytizing A*β* compared with resident microglia [53]. These studies highlighted the potential of transplantation of BM-derived progenitor cells from healthy individuals into AD patients as a future therapeutic approach. However, the number of such explorations is still limited and the therapeutic effects still need to be improved.

## 4. Brain Tumor

Brain tumor is a mass of abnormal cells in the brain which can be benign (cancerous) or malignant (noncancerous). The tumor can either originate from the brain or disseminate into the brain from other organs. There are many types of brain tumors; the most prevalent ones include intracranial metastases from other cancerous tissues, meningiomas, gliomas, and glioblastoma [66]. The most commonly seen brain tumor is glioma, both in children and in adults [67], which is categorized into four different grades based on severity [68]. In addition to cancer cells, glioma tissue also contains noncancer cells, including resident microglia and macrophages derived from circulating blood monocytes. These cells can comprise 30%-50% of the cellular content of the tumor [67] and contribute significantly to tumor microenvironment. There are multiple genetically engineered and viral vector-mediated transduction mouse models of human glioma as reviewed in [69]. These rodent models significantly facilitated the study of oncogenesis of brain tumors.

The tumor-associated macrophages (TAMs) may either be monocyte derived or microglia origin; a study using florescence reporter mice under control of CX3CR1 and CCR2 promoters showed that CX3CR1^low^CCR2^hi^ monocytes were recruited to the glioblastoma and transitioned into CX3CR1^hi^CCR2^low^ macrophages and CX3CR1^hi^CCR2^−^ microglia-like cells [70]. The authors further showed that a majority of TAMs are monocyte derived (around 85%) with the remaining 15% being resident microglia origin. Considering the majority of studies did not clearly separate these two populations, in this section, we will use the term TAMs with the assumption that a majority or at least a significant portion is monocyte derived.

The CCL2/CCR2 signaling is reported to be crucial for the development of TAMs and promotion of tumor growth in a rat glioma model [71]. The macrophages within the glioma normally express M2 markers, and their number positively corelated with histological grade of glioma [72]. However, glioma cell line expresses MCP-3/CCR7 instead of MCP-1/CCL2; moreover, MCP-3 but not MCP-1 [73] level corelated with the number of tumor-infiltrating macrophage in tissues from human patients. Another chemokine SDF-1/CXCR12 which was produced by tumor cells seems involved in the accumulation [74] of TAMs into areas of hypoxia. It should be noted that the blockade of CSF-1R signaling in vivo can reduce the number of TAMs and promote glioblastoma invasion in a mouse model. Whether this signaling works on resident macroglia or monocyte-derived macrophage or both needs to be further studied [75].

The majority of studies on TAMs indicated that this cell type is detrimental in many ways, including promoting tumor growth and invasion [68]. Glioma-associated peripheral blood monocytes may support immunosuppression and promote growth of malignant glioma by releasing unusually high amounts of EGF [76]. Human monocytes exposed to glioma cells develop myeloid-derived suppressor cell- (MDSC-) like properties with suppressive functions [77]. A study using a mouse model showed that when glioma cells were implanted together with monocytes, tumors grew faster with increased MDSCs and reduced CD8^+^ T cells [78]. Enrichment of microglia/macrophage-related genes is associated with shorter survival in adult glioblastoma tumors but not in children implying the detrimental role of macrophages [79]. M-CSF seems to play an important role during the development of glioma-associated M2 macrophages [72]. Although the prevailing literature indicated the tumor-promoting detrimental role of monocyte/macrophages, some occasional studies reported the relation of macrophages positively corelates with survival with unknown mechanism. A clinical study of IDH1R132H-non-mutant glioblastoma showed higher amount of glioma-associated macrophages in the vital tumor core was associated with increased patient survival [80].

Considering the significant tumor-promoting effects in most cases, targeting the TAMs has been used as a therapeutic strategy such as targeting CSF-1R [68]. Although monocytes/macrophages are seemly harmful in most tumors, they do demonstrate tumor-killing effects in certain situations [81]. In addition, considering the dramatic tumor-infiltrating effect of monocytes, there are studies utilizing modified monocytes to deliver drugs to tumors [82].

## 5. Intracerebral Hemorrhage

Intracerebral hemorrhage is a life-threatening condition which is normally associated with subsequentially brain inflammation. Monocytes are rapidly mobilized after the onset of intracerebral hemorrhage. In this situation, monocytes play a role contributing to the inflammation which is detrimental for disease progression; on the other hand, monocyte can be protective due to involvement in the clearance of the hematoma and the functional recovery after intracerebral hemorrhage [83].

The recruited monocytes infiltrate and differentiate into macrophages within 12 hours post intracerebral hemorrhage in a mouse model [84]. The Ly6C^hi^ monocytes are the dominant leukocytes recruited to the brain which outnumber other cell types and contribute to the proinflammatory environment by secreting TNF-*α* [85]. CCR2 knockout mice and chimeric mice with WT somatic cell and CCR2 knockout bone marrow-derived cells showed reduced number of Ly6C^hi^ monocytes and mitigated disease outcome with less inflammation after intracerebral hemorrhage [85]. Neutrophils were reported to enhance the recruitment of monocyte into the hemorrhagic brain, but the exact mechanisms are not clear [86].

The role of monocyte and monocyte-derived macrophages is not consistent in different models. While Hammond et al. reported the detrimental role of inflammatory monocytes in short-term studies [85], many others report macrophages can prevent hemorrhagic infarct transformation in a mouse stroke model by mediating neuroprotection and the repair of ischemic neurovascular tissues [87, 88]. In this study [87], mice were treated with clodronate liposomes at days 1 and 2 after stroke induction and showed an increased rate of visible hemorrhage on days 3 and 7. The same result can be repeated using CD11b-DTR mice or targeting of CCR2 in bone marrow-derived cells suggesting monocyte-derived macrophages were essential for establishing integrity after stroke [89].

Comparing to the complex roles of Ly6C^hi^ monocytes, Ly6C^low^ monocytes do not play a noticeable role in acute or recovery phase after intracerebral hemorrhage suggesting this monocyte subset may not be a therapeutic target [90].

Similar to monocyte-derived macrophages during stroke, macrophages developed during intracerebral hemorrhage can also switch its phenotypes between the inflammatory M1 status and M2 protective/reparative status. A study showed IL-4 treatment at the early stage can potentially promote neurofunctional recovery by enhancing M2 transition [91]. The engulfment of erythrocytes with exposed phosphatidylserine modulates macrophages towards recovery after intracerebral hemorrhage [83]. Mice without receptor tyrosine kinases AXL and MERTK have reduced hematoma clearance [83]. It should also be noted that peroxisome proliferator-activated receptor gamma (PPAR*γ*) may be an important mediator for activation of M1 to M2 transition [92]. PPAR*γ* activators rosiglitazone significantly increased the expression of PPAR*γ*-regulated gene catalase and CD36, while such treatment inhibited the proinflammatory genes including TNF-*α*, IL-1*β*, MMP-9, and iNOS. This is associated with reduced extracellular H(2)O(2) level, and neuronal damage. Conversely, phagocytosis capacity was significantly inhibited by PPAR*γ* gene knockdown or CD36-neutralizing antibody but can be enhanced by exogenous catalase [92].

## 6. Multiple Sclerosis

Multiple sclerosis is an inflammatory-demyelinating disease of the central nervous system caused by abnormal immune-mediated response. The MS lesions contain a substantial accumulation of monocytes [93]. The experimental autoimmune encephalomyelitis (EAE) model is the most commonly used animal model to study MS, although this model is not perfect [94]. Monocyte infiltrates the MS lesions and contributed to the progression to the paralytic stage of EAE [95, 96]. The monocytes infiltrated will differentiate into inflammatory macrophages which are indistinguishable from resident microglia. CCR2 plays a crucial role in the recruitment of monocytes and the disease progression in this EAE model [95, 97] implying the Ly6C^hi^ origin of the monocytes. Mice without CCR2 are resistant to EAE [98] which is associated with reduced in T cell number [97]. In addition, IL-1*β* is required for the transmigration of Ly6C^hi^ monocytes across endothelial cells of the central nervous system [99]. Interestingly, mouse lacking CCR4 is also reported to be resistant in EAE models [100]; CCR4^+^ DCs, but not macrophages, of bone marrow origin are the cellular mediators for EAE development through the release of GM-CSF and IL-23 [100]. Considering the disease-promoting role of monocytes, targeting the inflammatory monocytes may be of therapeutic significance.

Less studies are focused on nonclassical monocyte. Although the number of monocytes in MS blood is normal [101], it is found that MS patients have increased circulating CD14^+^16^++^ nonclassical monocytes but lower classical monocytes comparing to healthy controls [102]. Moreover, a study of clinical samples revealed the number CD16^+^ intermediate monocyte subset is reduced in peripheral blood of MS patients, although this subset is a dominant monocyte subset in the CSF under noninflammatory conditions [103]. CD16^+^ monocytes are seen within active MS lesions in a perivascular location and are shown to promote CD4^+^ T cell in a transwell migration assay of the blood-brain barrier model [103]. The authors suggest that this subset may contribute to the breakdown of the blood-brain barrier. However, the exact roles of these subsets need further studies.

## 7. Insomnia

Insomnia, a sleep disorder caused by factors ranging from emotional issues to physical problems, is profoundly but closely associated with the immune system. Sleeping wellness also have an effect on the monocyte count in the circulation [104], and sleep-deprived individuals are reported to have increased monocyte counts [105]. Mice subjected to sleep fragmentation generated more Ly6C^hi^ monocytes which contributed to larger atherosclerotic lesions [106]. Monocyte is affected by circadian clocks of the host [107]; disturbance of sleep leads to altered circadian rhythms of immune functions [108]. In a mouse model, Ly6C^hi^ inflammatory monocyte subset exhibits diurnal variation which controls their trafficking to inflammation sites and better protects against bacterial infections [107]. Moreover, the circadian gene *Bmal1* is shown to play a critical role in controlling of the rhythmic variation of monocyte number [107]. Mice sleeping 6 hours a day have more circulating monocytes comparing mice without sufficient sleep [109]. These increases of monocytes are only partially dependent upon CCR2 signaling but not intercellular adhesion molecule 1 (ICAM-1) or lymphocyte function-associated antigen 1 (LFA-1). The clock gene *Arntl* whose expression is suppressed by sleeping orchestrates the increase of monocytes into the circulation [109]. One reason for the effect of sleep on monocyte number is likely associated with cortisol production which peaks 30 min after waking and falls thereafter [105]. Cortisol is the endogenous substance which works in a fashion like glucocorticoid drugs that are known to be anti-inflammatory and can reduce monocyte number [110].

Sleeping affects not only the number of monocytes but also their functions. Studies have shown that monocyte from mice with sleep is associated with enhanced production of reactive oxygen species and improved defense against bacterial infections [109]. Sleeping can also increase the number of IL-12-producing monocytes and reduce the number of IL-10-producing monocytes [111] such as promoting a more inflammatory status in aid for immune protection [112]. This result is consistent with another study showing sleep was associated with a striking increase in the number of pre-mDC producing IL-12, which is a main inducer of Th1 responses [104]. This study also reported a substantial reduction in the number of CD14^dim^CD16^+^ monocytes.

However, considering the complex causes of insomnia, current studies on animal models mostly adopted the sleep deprivation model which hurdled the exploration of the actual role of monocytes during clinical settings. Whether monocyte plays a role during the onset of insomnia is unclear; moreover, the changes in monocyte number and function induced by insomnia on the immunity against infection, autoimmune disease, and other related diseases still need further investigation.

## 8. Other Brain Disorders

Many other brain disorders also significantly affect the patients' quality of life; these diseases include mental disorders, Parkinson's disease, and seizure disorders. The basic research of the role of monocytes on these diseases is not as widely studied as the above discussed disorders, but still, there are studies exploring the contributions of monocytes to these diseases. For example, in a status epilepticus (continuous seizure) model, it is shown that Ly6C^hi^ monocytes also invade the brain after seizures in a CCL2-dependent manner and contribute to the inflammatory environment. Inflammation in this case is detrimental, and blocking the monocyte recruitment to the brain is beneficial to the host [113]. This event is associated with the leakage of blood-brain barrier and the activation of microglia [113].

Multiple studies have indicated the association between monocyte activity and mental disorders [114], and patients with schizophrenia showed increased monocytes in the circulation and in the cerebrospinal fluid according to some studies [114]. The role of these monocytes during the progression of mental disorders also need more investigation.

## 9. Concluding Remarks

This review focused on the role of monocytes during the common brain disorders, especially by focusing on ischemic stroke, Alzheimer's disease, brain tumor, multiple sclerosis, intracerebral hemorrhage, and insomnia. Monocyte recruitment is commonly seen and sometimes dramatically accumulated in all these diseases. A majority of research has been focused on the role of Ly6C^hi^ monocytes, while its counterpart, the Ly6C^low^ monocytes, is less involved. The functions of monocytes are normally complexed and significantly depend on the disease model and disease stage. This is very conceivable since monocyte is a cell type with dramatic plasticity and can switch its status between inflammatory to tissue remodeling. Most importantly, monocytes have the ability to infiltrate the CNS at both early and late stages of the disease. This special property makes it a promising target for immunotherapy or immunoregulation. Studies have been ongoing to utilize this cell as vehicle to deliver drugs to the brain. The studies on the monocyte recruitment, differentiation, and function in these diseases will continue to unveil the process of disease progression and design of therapeutic strategies.

## Abbreviations

AD: Alzheimer's disease
ICH: Intracerebral hemorrhage
CCR2: C-C chemokine receptor type 2
MCP-1: Monocyte chemoattractant protein-1
PPRs: Pattern recognition receptors
CX3CR1: C-X3-C motif chemokine receptor 1
Nr4a1: Nuclear Receptor Subfamily 4 Group A Member 1
MCAO: Permanent middle cerebral artery occlusion
NFT: Neurofibrillary tangles
APP: Amyloid precursor protein
RAGE: The receptor for advanced glycation end products
VLA4: Very late antigen 4
DAMPs: Damage-associated molecular patterns
NALP3: NACHT, LRR and PYD domain-containing protein 3
TAM: Tumor-associated macrophage
A*β*: Amyloid beta
LRP: Low-density lipoprotein receptor-related protein
SCA: Scavenger receptor A
PECAM-1: Platelet endothelial cell adhesion molecule 1
BM: Bone marrow
EGF: Epidermal growth factor
TNF-*α*: Tumor necrosis factor alpha
SDF-1: Stromal cell-derived factor 1
MDSC: Myeloid-derived suppressor cells
EAE: Experimental autoimmune encephalomyelitis.
